# Instance-Level Contrastive Learning for Weakly Supervised Object Detection

**DOI:** 10.3390/s22197525

**Published:** 2022-10-04

**Authors:** Ming Zhang, Bing Zeng

**Affiliations:** School of Information and Communication Engineering, University of Electronic Science and Technology of China, No. 2006, Xiyuan Avenue, West Hi-Tech Zone, Chengdu 611731, China

**Keywords:** weakly supervised object detection, instance-level contrastive learning, memory-aware instance mining, memory-aware proposal sampling

## Abstract

Weakly supervised object detection (WSOD) has received increasing attention in object detection field, because it only requires image-level annotations to indicate the presence or absence of target objects, which greatly reduces the labeling costs. Existing methods usually focus on the current individual image to learn object instance representations, while ignoring instance correlations between different images. To address this problem, we propose an instance-level contrastive learning (ICL) framework to mine reliable instance representations from all learned images, and use the contrastive loss to guide instance representation learning for the current image. Due to the diversity of instances, with different appearances, sizes or shapes, we propose an instance-diverse memory updating (IMU) algorithm to mine different instance representations and store them in a memory bank with multiple representation vectors per class, which also considers background information to enhance foreground representations. With the help of memory bank, we further propose a memory-aware instance mining (MIM) algorithm that combines proposal confidence and instance similarity across images to mine more reliable object instances. In addition, we also propose a memory-aware proposal sampling (MPS) algorithm to sample more positive proposals and remove some negative proposals to balance the learning of positive-negative samples. We conduct extensive experiments on the PASCAL VOC2007 and VOC2012 datasets, which are widely used in WSOD, to demonstrate the effectiveness of our method. Compared to our baseline, our method brings 14.2% mAP and 13.4% CorLoc gains on PASCAL VOC2007 dataset, and 12.2% mAP and 8.3% CorLoc gains on PASCAL VOC2012 dataset.

## 1. Introduction

Object detection is a fundamental task in computer vision, which requires to identify object categories and use bounding boxes to locate their complete region positions. With the development of convolutional neural network (CNN) [1,2,3], some object detection methods [4,5,6,7,8,9,10,11,12,13], such as Fast R-CNN [4], Faster R-CNN [5], SSD [6] and YOLO [7], have made significant progress. However, these methods require fully supervised information, i.e., instance-level annotations, which are time-consuming and labor-intensive to label. To reduce the burden of annotations, weakly supervised object detection (WSOD) removes bounding boxes and only requires image-level annotations, i.e., image tags, to indicate whether object categories are present in an image.

**Figure 1:**
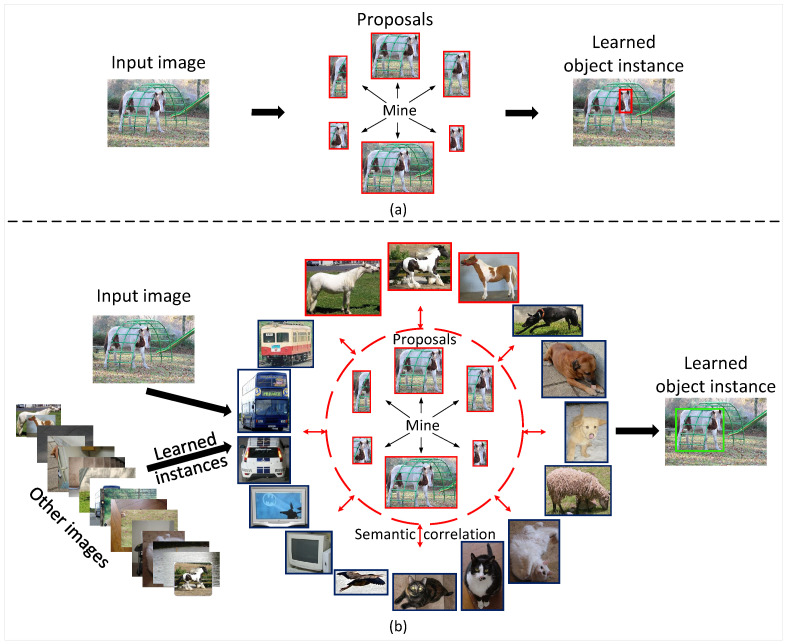
Illustration of our motivation through data flow. (**a**) Most WSOD methods usually learn object instances from region proposals of current input image. (**b**) Our method establishes instance correlations with other images to guide instance representation learning for the current input image.

Due to lack of object bounding box position supervision, most current WSOD methods [14,15,16,17,18,19,20,21,22,23,24,25,26,27] use multiple instance learning (MIL) [28] to mine object instances from pre-generated proposals, and treat them as pseudo instance-level annotations to train weakly supervised detectors. However, these methods only focus on a single image to learn object representations without considering the internal relevance of various object instances across images. When there are object appearance variations in the complex diverse image scenes, it is easy to cause false detection. For example, when a horse is occluded in Figure 1, it focuses more on local feature representation, which is not enough to represent the whole object, resulting in the learned object instance only covering the head of the horse.

To deal with the problem, we propose an instance-level contrastive learning (ICL) framework to store reliable instance representations from all learned images, and utilize contrastive learning [29] mechanism to explicitly establish semantic correlations with other image instances. It attempts to enhances the discriminative and robustness of object instance representations in the current input image, pulling it close to the instance representations of same class from all training images and pushing it away instance representations of different classes. As shown in Figure 1b, owning to instance correlation with other images, our method can effectively learn the instance representation for the whole horse.

To sufficiently represent diversity instances in all training data, we next propose an instance-diverse memory updating (IMU) algorithm. It mines reliable instance representations from proposal features and builds a memory bank with multiple representation vectors for each class to store them based on similarity, where background information is also consider to enhance foreground representations. Based on the memory bank, we further propose a memory-aware instance mining (MIM) algorithm. Unlike most methods [14,15,20,21,22,26,27] that mine object instances only based on proposal confidence, we also compute the similarity with stored diverse instances to evaluate the completeness of proposals to mine more reliable object instances. Instead of selecting the top-scoring proposal as an instance, we also consider the multi-instance case to mine more instances in an image. During the training process of weakly supervised object detectors, we propose a memory-aware proposal sampling (MPS) algorithm to alleviate the imbalance problem between positive and negative samples. According to the similarity with instance representations, we select more positive proposals to increase the number of positive samples. Based on the similarity with the background information, we remove some negative proposals with low similarity to reduce the number of negative samples.

To verify the effectiveness of our method, we conduct extensive experiments on the PASCAL VOC2007 and VOC2012 datasets, which are widely used for weakly supervised object detection. In this paper, we adopt typical WSOD method OICR [15] as our baseline, which can be easily embedded into our ICL framework to further improve the performance. On PASCAL VOC2007 dataset, our method improves detection performance and localization accuracy by 14.2% and 13.4% in terms of mAP and CorLoc, respectively. On PASCAL VOC2012 dataset, our method improves performance by 12.2% mAP and 8.3% CorLoc.

The contributions of this paper are summarized as follows:

- We propose an instance-level contrastive learning (ICL) framework to guide the weakly supervised detector to learn instance representations. To the best of our knowledge, we are the first to explore contrastive learning in weakly supervised object detection.
- We propose an instance-diverse memory update (IMU) algorithm to store reliable instance representations into a memory bank, where multiple representation vectors are used in each class to maintain the diversity of instance representations.
- With the help of memory, we further propose a memory-aware instance mining (MIM) algorithm to efficiently mine object instances by combining proposal confidence and instance similarity.
- With the help of memory, we also propose a memory-aware proposal sampling (MPS) algorithm to alleviate the imbalance between positive and negative samples by finding more positive proposals and removing some unreliable negative proposals.

## 2. Related Work

In this section, we present the two most relevant to this paper, weakly supervised object detection and contrastive learning.

### 2.1. Weakly Supervised Object Detection

Since Hakan Bilen and Andrea Vedaldi proposed a weakly supervised deep detection network (WSDDN) [20] to combine MIL and CNN into an end-to-end network, most WSOD methods follow [14,15,16,17,18,19,21,22,26,27] this pipeline to train the weakly supervised detector. In MIL, an image is treated as a bag of proposals. If the image contains an object class, this bag is labelled as positive bag, i.e., at least containing one object instance of this class, otherwise labelled as negative bag. Due to lack of instance-level annotations, MIL is tend to get stuck in a local optimum to locate the most representative part of target objects. Subsequently, most researchers have proposed promising approaches to alleviate this problem. For instance, Kantorov et al. [14] proposed additional and contrastive context-aware guidance models to improve localization by using the surrounding contextual region of proposals. Tang et al. [15] proposed an online instance classifier refinement (OICR) method that uses spatial correlations between proposals to refine mined instances. Wan et al. [21] proposed continuation multiple instance learning (C-MIL) to alleviate the problem that MIL is prone to falling into local optima, which uses some smooth loss function to approximate the original non-convex loss function. Lin et al. [22] proposed object instance mining (OIM) framework to build spatial and appearance graphs of proposals to mine all possible object instances. Furthermore, some methods [16,17,18,19] introduce segmentation information to assist instance mining. Shen et al. [16] proposed a recurrent guidance strategy for weakly supervised detection and segmentation, where the detection module generates seeds for semantic segmentation and the segmentation module provides prior information for object detection. Yang et al. [17] proposed an objectness consistent representation method to exploit segmentation map to mine more high-quality proposals. Wei et al. [18] used segmentation context information around proposals to discover tight object bounding boxes. Li et al. [19] leveraged the segmentation map to reweight proposals scores. However, these methods only consider information from a single image, which are difficult to deal with diverse object instances. In this paper, our method explores the semantic correlation beyond the input image to assist object instance mining.

### 2.2. Contrastive Learning

Contrastive learning [29] has been widely used in unsupervised representation learning (e.g., SimCLR [30] and MoCo [31]), which compares positive and negative pairs to compress together different view representations of the same image and separate view representations of different images. In addition, contrastive learning-based methods [32,33,34,35] have also achieved promising performance in other vision tasks. For instance, Yan et al. [33] proposed a semantics-guided contrastive network that introduces contrastive learning into zero-shot object detection to transfer available semantic information for unseen classes. Wu et al. [34] proposed a contrastive learning-based robust object detection algorithm to detect objects under smoky conditions, which applies contrastive learning to maximize the consistency between different augmented views of the same smoke image. Li et al. [35] introduced contrastive learning into remote sensing image semantic segmentation to learn global and local image representations. However, these methods are difficult to directly apply to WSOD that learns the detector based on image-level annotations. In this paper, we introduce contrastive learning into weakly supervised object detection and propose an instance-level contrastive learning framework. To our best knowledge, we are the first to explore contrastive learning for weakly supervised object detection.

## 3. Method

In this section, we first describe our instance-level contrastive learning (ICL) framework in detail. Then, we present the instance-diverse memory updating (IMU) algorithm, memory-aware instance mining (MIM) algorithm and memory-aware proposal sampling (MPS) algorithm.

### 3.1. Instance-Level Contrastive Learning

**Figure 2:**
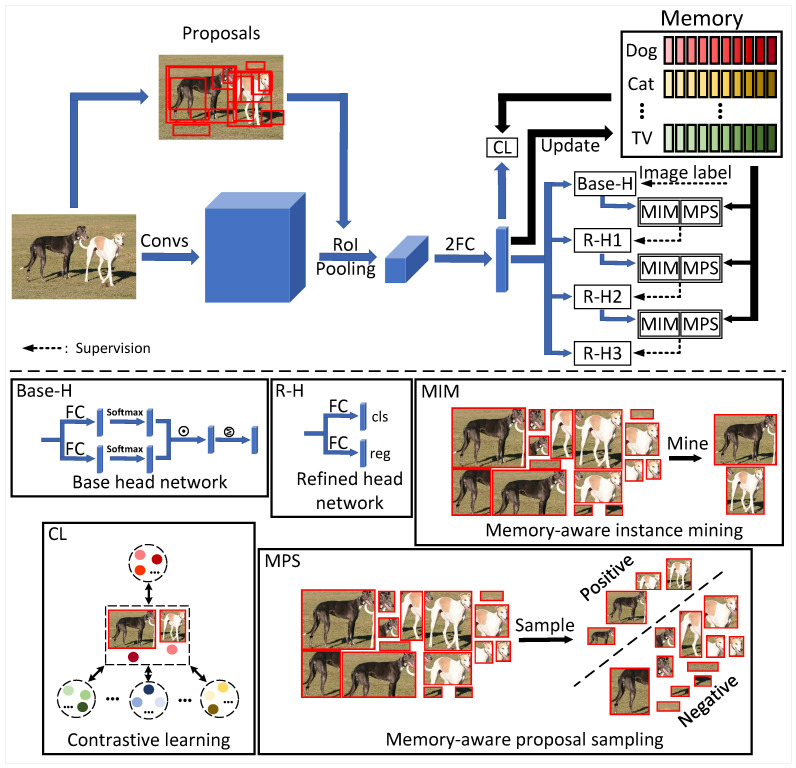
The pipeline of instance-level contrastive learning (ICL) framework. The upper part shows the overall network structure, where only the blue arrows backpropagate the gradients. There are one base head (Base-H) and three refined heads (R-H1, R-H2, R-H3). The base head is supervised by image labels, while each refined head is supervised by the previous parallel head. Dashed arrows indicate the supervision information. The detailed network structure of these heads can be found in the lower boxes, where each box corresponds to a submodule in the pipeline. Three refined heads have the same network structure but do not share parameters. The processes of memory-aware instance mining (MIM) algorithm, memory-aware proposal sampling (MPS) algorithm and contrastive learning (CL) are also shown in boxes.

In Figure 2, we present the pipeline of the instance-level contrastive learning (ICL) framework. Given an input image *I* and the corresponding proposals *R* generated by the proposal generation methods [36,37,38], we first extract image features $F_{I}$ using convolutional neural network. Based on the pre-generated proposals *R*, we convert image features $F_{I}$ to proposal features $F_{R}$ through a RoI-pooling layer, and use two fully connected (FC) Layers to obtain proposal vector representations $F_{V}$. By mining reliable instance representations from $F_{V}$, we then perform contrastive learning (CL) and store them in the memory bank *M*. In addition, $F_{V}$ is fed into several parallel detection heads, where a base head is supervised by the image label $Y={\left[y_{1},y_{2},\ldots ,y_{C}\right]}^{T}\in R^{C\times 1}$ and *K* refined heads are supervised by the output results of previous heads, where *C* is the number of classes. In this paper, we set K=3 to be the same as our baseline method [15].

Unsupervised representation learning [30,31] performs contrastive learning by augmenting image to different views, where views of the same image are pulled closer and views of different images are pulled apart. In this paper, we introduce the contrastive learning of object instance representations to guide the detector to learn the entire representation of the instance. Specifically, we first denote all outputs of refined heads as $\left(\left\{\varphi^{1},\varphi^{2},\ldots ,\varphi^{K}\right\},\left\{t^{1},t^{2},\ldots ,t^{K}\right\}\right)$. To mine more reliable instances, we average these outputs to obtain the proposal scores $\varphi =\frac{1}{K}\sum_{k=1}^{K}\varphi^{k}$ and the bounding box coordinate offsets $t=\frac{1}{K}\sum_{k=1}^{K}t^{k}$. Applying the coordinate offset *t* to transform *R*, we obtain the transformed proposal *P*. Then, we exploit non-maximum suppression (NMS) to mine as many object instances as possible. For a positive class *c*, we use NMS to gradually select object instances from the transpose proposal $P_{c}$ according to the proposal score $\varphi_{c}$ from high to low, and remove redundant proposals. Then, we set a score threshold $T_{1}$ to obtain more reliable object instances *D*, and extract the corresponding instance feature representations $F_{D}$ from $F_{V}$. The detailed procedure can be seen in Algorithm 1. For each mined instance representation $q\in F_{D}$, we utilize the memory instance representation *M* including all training data to assist each instance learning in current image. Assume that a positive representation from memory bank $k_{+}\in M$ represent the same class as *q*, and a negative representation from memory bank $k_{-}\in M$ represent different classes. Then, we use the contrastive loss [39] to pull *q* close to $k_{+}$ of the same class while pushing it away from negative keys $k_{-}$ of other classes, and thus enhance the discrimination and generalization of current instance representation:

(1) $$L_{CL}=-\frac{1}{\left|D\right|}\sum_{q\in F_{D}}\varphi_{q}log\frac{exp(q\cdot k_{+}/\tau )}{exp\left(q\cdot k_{+}/\tau \right)+\sum_{k_{-}}exp\left(q\cdot k_{-}/\tau \right)},$$

where we take $\varphi_{q}$ as the loss weight and τ means the temperature hyperparameter.
**Algorithm 1** Instance representation mining algorithm.**Input: ** The pre-generated proposals *R*, the pre-defined score threshold $T_{1}$, the image label *Y*, the memory bank *M*, the outputs of instance refined heads $\left(\left\{\varphi^{1},\varphi^{2},...,\varphi^{K}\right\},\left\{t^{1},t^{2},\ldots ,t^{K}\right\}\right)$ and the proposal feature vectors $F_{V}$.   (I) average proposal scores $\varphi =\frac{1}{K}\sum_{k=1}^{K}\varphi^{k}$   (II) average coordinate offsets $t=\frac{1}{K}\sum_{k=1}^{K}t^{k}$   (III) obtain transformed proposals *P* by adding *t* to *R*   (IV) instance representations $F_{D}=⌀$ and the corresponding confidences $\varphi_{F_{D}}=⌀$   **For** c=0
**to**
C+1      **If** $y_{c}==1$
**or**
c==C+1         (1) $keep=NMS(P_{c},\varphi_{c})$         (2) $P_{keep}=P_{c}\left[keep\right]$         (3) $\varphi_{keep}=\varphi \left[keep\right]$         (4) $D_{c}=P_{keep}\left[\varphi_{keep}>T_{1}\right]$         (5) $\varphi_{D_{c}}=\varphi_{keep}\left[\varphi_{keep}>T_{1}\right]$         (6) $F_{D_{c}}=F_{V}\left[D_{c}\right]$         (7) $F_{D}=F_{D}\cup F_{D_{c}}$, $\varphi_{F_{D}}=\varphi_{F_{D}}\cup \varphi_{D_{c}}$
**Output:**
$F_{D}$, $\varphi_{F_{D}}$.


Subsequently, we describe the training of heads. The base head has two parallel branches. One branch uses an FC layer to generate a matrix $x^{c}\in R^{C\times |R|}$ (|R| is the number of proposals), which is then input to a class-wise softmax layer: ${\left[\sigma \left(x^{c}\right)\right]}_{ij}=\frac{e^{x_{ij}^{c}}}{\sum_{q=1}^{C}e^{x_{qj}^{c}}}$. In another branch, there is an FC layer and a proposal-wise softmax layer to generate another normalized matrix $\sigma (x^{r})$, where $x^{r}\in R^{C\times |R|}$ and ${\left[\sigma \left(x^{r}\right)\right]}_{ij}=\frac{e^{x_{ij}^{r}}}{\sum_{q=1}^{\left|R\right|}e^{x_{iq}^{r}}}$. Then, element-wise matrix multiplication is performed on these two matrices to generate proposal scores $x^{R}=\sigma \left(x^{c}\right)⊙\sigma \left(x^{r}\right)$. Finally, the image class score is calculated by summing all proposal scores: $\phi =\sum_{r=1}^{\left|R\right|}x^{R}$. According to the image label $Y={\left[y_{1},y_{2},...,y_{C}\right]}^{T}$, the loss of base head is computed by Equation (2).

(2) $$L_{b}=-\sum_{c=1}^{C}\left\{y_{c}\log\phi_{c}+\left(1-y_{c}\right)\log\left(1-\phi_{c}\right)\right\}.$$

For *K* refined heads, their training process is consistent. Specifically, for the $k^{th}$ head, there is a classifier and a regressor. In the classifier, an FC layer and a class-wise softmax are used to generate proposals scores $\varphi^{k}\in R^{\left(C+1)\times |R\right|}$, where C+1 means background is included. In the regressor, an FC layer is used to produce the coordinate offsets of proposals $t^{k}\in R^{4C\times |R|}$, where 4 means the dimension of coordinate offsets $\left(x_{1},y_{1},x_{2},y_{2}\right)$. In order to generate their supervision, we first use the memory-aware instance mining (MIM) algorithm to mine multiple representative object instances $B^{k}$ based on the score outputs and offset outputs of previous head $\left(\varphi^{k-1},t^{k-1}\right)$ and memory bank *M*. The details can be seen in Section 3.3. Then, we use the memory-aware proposal sampling (MPS) algorithm of Section 3.4 to sample negative and positive proposals $\left(R_{pos},R_{neg}\right)$ from proposals *R* and assign labels for these proposals. For a positive class *c*, if a proposal *p* is selected as positive sample, *p* is labeled as class *c*, i.e., $y_{c,p}^{k}=1$. All negative proposals $R_{neg}$ are labeled as background class C+1. In this way, the classifier can be trained by a cross entropy loss:

(3) $$L_{cls}^{k}=-\frac{1}{|R_{pos}\left|+\right|R_{neg}|}\sum_{p\in R_{pos}\cup R_{neg}}\sum_{c=1}^{C+1}\varphi_{p}^{k}y_{c,p}^{k}\log\varphi_{c,p}^{k},$$

where we also use the confidence $\varphi_{p}^{k}$ as the loss weight. For the regressor, only positive proposals $R_{pos}$ are used to calculate loss by the smooth L1 loss [4]:

(4) $$L_{reg}^{k}=\mathrm{smoothL}1\left(t^{k},T^{k}\right),$$

where $T^{k}$ is the supervision of coordinate offsets.

In summary, our ICL framework can be end-to-end trained in Equation (5).

(5) $$L=L_{CL}+L_{b}+\sum_{k=1}^{K}\left(L_{cls}^{k}+L_{reg}^{k}\right).$$

### 3.2. Instance-Diverse Memory Updating Algorithm

In order to enable the network to memory the instance representations from previous training images, we first initialize $M\in R^{(C+1)\times N\times L}$, where *N* means the number of stored instance feature representations in each class and *L* represents the length of the feature vector $F_{V}$. Since instances of the same class differ in size, shape, and appearance, we use multiple feature vectors to store richer instance representations instead of a single vector. We first use the Algorithm 1 to obtain some reliable instance representations $F_{D}$ from $F_{V}$. For each instance representation $f_{d,c}\in F_{D}$, we calculate the similarity between $f_{d,c}$ and with $M_{c}=\left\{f_{c,1},f_{c,2},\ldots ,f_{c,N}\right\}$ in Equation (6).

(6) $$S_{d,c}=\left|\right|f_{d,c}\left|\right|\times \left|\right|M_{c}^{T}\left|\right|,$$

where ||·||, × and T mean $L_{2}$ normalization, matrix multiplication and transpose, respectively. Then, we select the most similarity feature $f_{c,j}$ from $M_{c}$ in Equation (7) to maximize the assistance of the current instance.

(7) $$j=argmax\{S_{d,c}\},$$

Finally, we update the feature vector $f_{c,j}$ according Equation (8) for the instance contrastive learning of subsequent images.

(8) $$f_{c,j}=r*f_{c,j}+\left(1-r\right)*\varphi_{f_{d,c}}*f_{d,c},$$

where *r* is the momentum coefficient [31] and $\varphi_{f_{d,c}}$ is the confidence of instance representation, which aim to control the weight balance between previous instance representation and current representation. The whole process can be seen in the Algorithm 2.
**Algorithm 2** Instance-diverse memory updating algorithm.**Input:** The pre-generated proposals *R*, the pre-defined score threshold $T_{1}$, the image label *Y*, the memory bank *M*, the outputs of instance refined heads $\left(\left\{\varphi^{1},\varphi^{2},\ldots ,\varphi^{K}\right\},\left\{t^{1},t^{2},\ldots ,t^{K}\right\}\right)$ and the proposal feature vectors $F_{V}$.   (I) obtain reliable instance representations $F_{D}$ using Algorithm 1   **For** each representation $f_{d,c}$
**in**
$F_{D}$      (a) compute the similarity between $f_{d,c}$ and with $M_{c}$ in Equation (6)      (b) choose the most similarity feature $f_{c,j}$ from $M_{c}$ in Equation (7)      (c) update $f_{c,j}$ in Equation (8) **Output:** Updated memory *M*.


### 3.3. Memory-Aware Instance Mining Algorithm

With the help of memory bank *M*, we propose a memory-aware instance mining (MIM) algorithm to effectively mine some reliable object instances. Different from our baseline [15], which only selects the top-scoring proposal as pseudo instance annotations, we comprehensively consider the confidence of proposals and the similarity between proposal features and memory bank covering previous training data to effectively mine object instances. Specifically, we first calculate the similarity *S* between $F_{V}$ and *M* according to Equation (9).

(9) $$S=\left|\right|F_{V}\left|\right|\times \left|\right|M^{T}\left|\right|.$$

Then, we select the highest similarity along the *N* feature vectors and apply the class-wise softmax to generate memory-base confidence $\varphi_{M}$ through Equation 10.

(10) $$\varphi_{M}=softmax\left(\underset{N}{max}\left\{S\right\}\right).$$

For the $k^{th}$ branch in instance refinement heads, we further calculate the combination confidence $\psi^{k}$ in Equation (11).

(11) $$\psi^{k}=\varphi^{k}+\mu \varphi_{M},$$

where μ is the combination coefficient. Next, we use the NMS algorithm to remove redundant proposals and set a score threshold $T_{2}$ to remove unreliable proposals. In this way, we can obtain some reliable instances $B^{k}$. More details can be found in Algorithm 3.
**Algorithm 3** Memory-aware instance mining algorithm.**Input:** The pre-generated proposals *R*, the pre-defined score threshold $T_{2}$, the image label *Y*, the memory bank *M*, the outputs of $k^{th}$ instance refined head $\left(\varphi^{k},t^{k}\right)$ and the proposal feature vectors $F_{V}$.   (I) obtain transformed proposals $R_{t^{k}}$ by adding $t^{k}$ to *R*   (II) calculate the memory-based confidence $\varphi_{M}$ by Equation (10)   (III) compute the combination confidence $\psi^{k}$ with Equation (11)   **For** c=0
**to**
*C*      **If** $y_{c}==1$         (1) $keep=NMS(R_{t^{k}},\psi_{c}^{k})$         (2) $R_{1}=R_{t^{k}}\left[keep\right]$         (3) $\psi_{1}=\psi_{c}^{k}\left[keep\right]$         (4) $B^{k}=R_{1}\left[\varphi_{1}>T_{2}\right]$         (5) $\psi_{B^{k}}^{k}=\varphi_{1}\left[\varphi_{1}>T_{2}\right]$ **Output:**
$B^{k}$, $\psi_{B^{k}}^{k}$.


### 3.4. Memory-Aware Proposal Sampling Algorithm

After mining object instances, we further propose a memory-aware proposal sampling (MPS) algorithm to effectively sample positive and negative proposals from *R*. Some methods [15,16,17,18,19,26] simply divide *R* into two parts by computing the IoU with $B^{k}$: highly overlapped proposals are taken as positive samples, and the rest are taken as negative samples, while ignoring the imbalance of positive and negative samples with overwhelmingly negative proposals. To alleviate this problem, we leverage the memory bank to select more positive proposals and remove some unreliable negative proposals. We first calculate the IoU between $B^{k}$ and *R* to separate *R* into two parts $R_{1}^{k}$ and $R_{2}^{k}$ in Equation (12).

(12) $$\left\{\begin{array}{c} R_{1}^{k}=\left\{p\in R|\exists b\in B^{k},IoU\left(p,b\right)⩾0.5\right\}\\ R_{2}^{k}=\left\{p\in R|\forall b\in B^{k},0.1<IoU\left(p,b\right)<0.5\right\} \end{array}\right.$$

Then, we extract the feature representations $F_{R_{2}^{k}}$ of $R_{2}^{k}$ from $F_{V}$. For each positive class *c*, we compute the similarity between $F_{R_{2}^{k}}$ and $M_{c}$, and use Equation (13) to choose the most similar proposal $p_{c}$ into $R_{1}$.

(13) $$j=argmax(|F_{R_{2}^{k}}|\times |M_{c}^{T}|).$$

For the remaining proposals in $R_{2}^{k}$, we compute the similarity $S_{R_{2}^{k}}=\underset{N}{max}\left\{\right|F_{R_{2}^{k}}\left|\times \right|M_{c}^{T}\left|\right\}$ between $R_{2}^{k}$ and background information $M_{C+1}$ and sort $S_{R_{2}^{k}}$ according to similarity from high to low. Finally, We removed the last low-similarity 1/λ proposals from $R_{2}^{k}$ to obtain the negative samples. More details can be found in Algorithm 4.
**Algorithm 4** Memory-aware proposal sampling algorithm.**Input:** The pre-generated proposals *R*, the image label *Y*, the memory bank *M*, the mined object instance $\left(B^{k},\psi_{B^{k}}^{k}\right)$ and the proposal feature vectors $F_{V}$.   (I) positive samples $R_{pos}=⌀$, negative samples $R_{neg}=⌀$   (II) calculate $IoU(B^{k},R)$   (III) separate *R* into two parts $R_{1}^{k}$ and $R_{2}^{k}$ using Equation (12).   (IV) $R_{pos}=R_{pos}\cup R_{1}^{k}$   (V) extract feature representations $F_{R_{2}^{k}}$ from $F_{V}$   **For** c=0
**to**
*C*      **If** $y_{c}==1$         (1) calculate the similarity between $F_{R_{2}^{k}}$ and $M_{c}$         (2) select the most similar proposal $p_{c}$ from $R_{2}^{k}$ by Equation (13)         (3) $R_{pos}=R_{pos}\cup p_{c}$, $R_{2}^{k}=R_{2}^{k}/p_{c}$, $F_{R_{2}^{k}}=F_{R_{2}^{k}}/F_{p_{c}}$   (VI) $S_{R_{2}^{k}}=\underset{N}{max}\left\{\right|F_{R_{2}^{k}}\left|\times \right|M_{c}^{T}\left|\right\}$   (VII) sort $S_{R_{2}^{k}}$ from from high to low   (VIII) obtain $R_{neg}$ by removing the last low-similarity 1/λ proposals from $R_{2}^{k}$ **Output: **
$R_{pos}$, $R_{neg}$.


### 3.5. Test

After training, only the instance refined heads are used for testing. We perform the same operations as our baseline method [15]. We average the outputs of all refined heads to generate the final detection results.

## 4. Experiments

In this section, we first introduce experimental data and evaluation criteria, and elaborate on experimental details. Then we validate the advantages of our method by comparing with some recent methods. Finally, we conduct extensive ablation experiments to demonstrate the effectiveness of our method.

### 4.1. Datasets and Evaluation Measures

We conduct experiments on PASCAL VOC2007 [40] and VOC2012 [41] datasets, which are widely used in weakly supervised object detection setting [14,15,16,17,18,19,20,21,22,25,26,27]. In the PASCAL VOC2007 dataset, there are 9962 images belonging to 20 categories. These images are divided into three sets: *train*, *val*, *test*. According to the widely used WSOD setting, the *trainval* set (5011 images) is used for training. The PASCAL VOC2012 dataset has 22531 images split into *train*, *val* and *test* sets. The *trainval* set has 11540 images for training. It is important to note that all experiments have only image-level labels for training. For evaluation, there are two evaluation measures mean average precision (mAP [40]) and correct localization (CorLoc [42]). mAP is the standard PASCAL VOC protocol, which first computes the average precision (AP) for each class and then averages over all classes. AP for each class is obtained by calculating the area under the precision-recall curve. The mAP is used to evaluate performance on the *test* set. The second metric CorLoc is used to measure the localization accuracy of the *trainval* set. For each class, CorLoc is calculated as the ratio of images where at least one object is correctly localized. Both mAP and CorLoc are based on the PASCAL criterion. The object is considered to be successfully detected, when the intersection over union (IoU) between the ground-truth and predicted boxes is greater than 0.5.

### 4.2. Experimental Details

All experiments are performed on the Detectron2 (https://github.com/facebookresearch/detectron2) deep learning framework and 4 NVIDIA GTX 1080ti GPUs. Following our baseline method OICR [15], we use the the VGG16 [2] model as our backbone, pre-trained on the ImageNet dataset [43]. For pre-generated proposals, we use multiscale combinatorial grouping (MCG) method [38] to generate approximately 2000 proposals per image. During the training phase, we set the learning rate to 0.001 for the first 28 epochs and divide it by 10 for the next 12 epochs. In addition, we set the momentum and weight decay to 0.9 and 0.0005, respectively. The mini-batch size is set to 4, i.e., an image is run by one GPU. Regarding the data augmentation, we use 5 scales {480, 576, 688, 864, 1200} to randomly resize the shortest side of the image and make the longest side no more than 2000, where the random horizontal flips are also used. During the test stage, We average the output of all augmented data to generate final detection results. For Hyperparameters in our method, we set K=3, N=5, μ=0.1, 1/λ=1/4, and $T_{1}=T_{2}=0.5$. All settings in the PASCAL VOC2007 and VOC2012 datasets are the same.

### 4.3. Comparison with Other Methods

On PASCAL VOC2007 and VOC2012 datasets, we compare our method with some recent methods [14,15,16,17,18,19,20,21,22,25,26,27] to present our advantages.

For the PASCAL VOC2007 dataset, we present the detection performance (mAP) and localization accuracy (CorLoc) in Table 1 and Table 2, respectively. In terms of mAP, our method ICL achieves a detection performance of 55.4%, which brings a significant improvement (about 14.2%) compared to our baseline OICR [15] (41.2%). Our method also outperforms methods [16,17,18,19] that exploit segmentation information to learn instance representation. For example, compare with [17], our method has an advantage of about 4.8%. Furthermore, our method also has some improvements (about 1.9%) compared to recent methods SLV[27] and D-MIL [25]. In terms of CorLoc, our method ICL achieves 74.0% localization accuracy. Compared with our baseline (60.6%), our method improves the performance by about 13.4%. Compared to the segmentation-assisted method WS-JDS [16] or SDCN [19], our method improves the performance by more than 7.2%. In addition, our method also shows significant advantages (more than 3%) compared to recent methods SLV [27] and D-MIL [25].

For the PASCAL VOC2012 dataset, we show both detection performance (mAP) and localization accuracy (CorLoc) in the Table 3. Our method achieves 50.1% mAP and 70.4% CorLoc, which are 12.2% and 8.3% improvement over the baseline, respectively. Compare to some recent methods [25,27], our method also bring some gains. In terms of mAP, our method outperforms the methods [27] and [25] by 0.9% and 0.5%, respectively. In terms of CorLoc, our method brings gains of 1.2% and 0.3%, respectively. These results further demonstrate the effectiveness of our method.

### 4.4. Ablation Study

In this part, we conduct extensive experiments to further discuss the effects of main components of our method. Without loss of generality, all experiments are performed on the PASCAL VOC2007 dataset.

**Figure 3:**
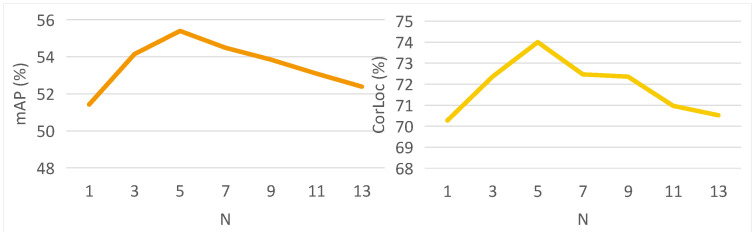
The effect of number of vectors N.

**The effect of IMU algorithm.** We first analyze the effect of IMU algorithm on our method ILC. In Table 4, after removing IMU, our method achieves 53.6% mAP and 72.9% CorLoc, respectively. There are 1.8% performance reduction and 1.1% accuracy reduction in terms of mAP and CorLoc, respectively, which proves the effectiveness of the instance-diverse memory updating algorithm. Furthermore, we analyze the effect of the number of feature vector *N* on the IMU algorithm in Figure 3. We can see that both mAP and CorLoc first increase and then decrease as *N* increases. When N is too small, the memory bank is difficult to store the diversity of instance representations well, and when N is too large, it is easy to cause there are internal differences during the learning of instance representations. In this paper, we recommend setting N=5 to balance the number of stored instance vectors.

**Figure 4:**
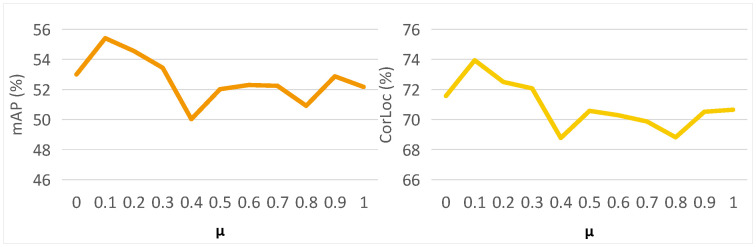
The effect of combination coefficient μ.

**The effect of MIM algorithm.** As shown in the Table 4, MIM brings 5.7% and 4.4% gains to ICL in mAP and CorLoc, which shows the effectiveness of memory-aware instance mining algorithm (MIM). In addition, we also analyze the effect of memory on MIM by setting different combination coefficients μ in Figure 4. During the change of μ from 0 to 1, we can see that both the detection performance and the localization accuracy are the highest at μ=0.1, which demonstrates that it is useful to introducing the similarity between memory features of previous training data and proposal features for mining effective instances. When μ becomes larger, the memory from previous images may hinder the learning of new instances from the current image, resulting in performance degradation. Therefore, we set μ=0.1 in this paper.

**The effect of MPS algorithm.** Removing MPS from ICL, our method achieves 52.9% detection performance (mAP) and 71.4% localization accuracy (CorLoc). There are 2.5% and 2.6% reductions in mAP and CorLoc, respectively, which shows the effectiveness of the memory-aware proposal sampling algorithm. In addition, we analyze the effect of the removal coefficient 1/λ on MPS in Table 5. We achieve the best performance when 1/λ=1/4. Continuing to increase 1/λ may remove too many negative samples and affect the training of the detector. When 1/λ is too small, it cannot achieve the purpose of balancing positive and negative samples.

**The performance of COCO metrics.** In Table 4, we also analyze the contribution of each component under the COCO metrics [44]. The performance of each component on AP, AP${}_{50}$, and AP${}_{75}$ is similar to that under the PASCAL metrics. For AP${}_{S}$, AP${}_{M}$ and AP${}_{L}$, the objects are divided into three sizes of small, medium and large for evaluation. Our method ICL can achieve the best performance on large objects, while removing MPS algorithm can achieve better performance on small and medium objects. Compared with small and medium-sized objects that are difficult to perceive, MPS algorithm is more conducive to sampling region proposals of large objects.

**Figure 5:**
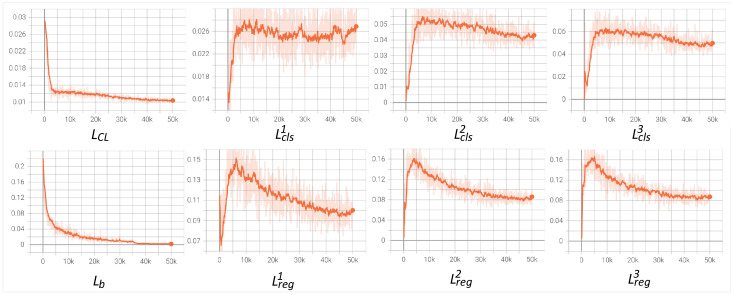
Training loss.

**Figure 6:**
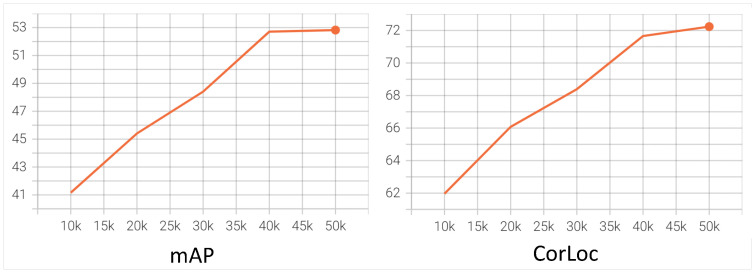
Model performance during the training process. Evaluations are performed every 10K without data augmentation.

**The analysis of training process.** In Figure 5, we further provide training loss curves to verify the rationality of our method. We can see that the loss curves of refined heads rise first and then decrease to convergence. The rising phase of the loss is due to the weight, which is the confidence of mined object instances. At the beginning of training, the low discrimination of the model makes the confidence of object instances very low (almost close to 0). As model capabilities increase, the confidence starts to increase and so does the loss. Since confidence range is [0,1], the loss will reach the maximum value, and finally start to decrease due to the enhanced model generalization until it converges. In Figure 6, we also provide the performance of the model during training. Both mAP and CorLoc continue to increase, which further demonstrates the effectiveness of our method.

**Figure 7:**
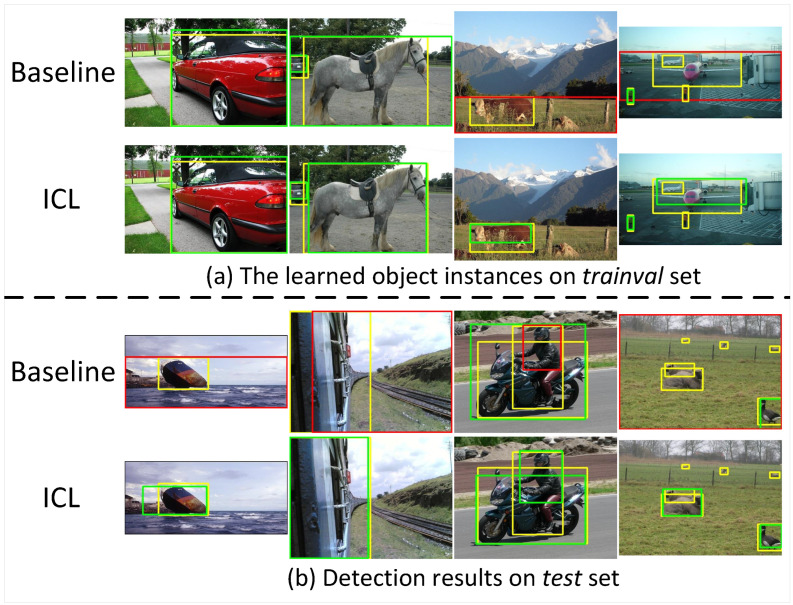
Qualitative results on PASCAL VOC2007 dataset. Yellow boxes mean ground truths. Green and red boxes represent correct and failed cases, respectively.

**Qualitative results.** In Figure 7, we provide qualitative results to more intuitively compare the proposed ICL with our baseline. On the *trainval* set of PASCAL VOC2007 dataset, we compare the learned object instances in Figure 7a. For the simple image in the first column, the baseline method can learn effective information about the car well. For the horse in the second column and the cow in the third column, when the foreground and background are relatively similar or the objects are occluded, the instances learned by the baseline method may contain more background. Our method ICL can learn more reliable object instances guided by instance correlations. On the *test* set, we compare the detection results in Figure 7b. Since the baseline method is more easily disturbed by background information during the training process, its detection results also contain more background information, such as the first two columns. Our method can better locate the boundary of the object. When there is interaction between objects, such as the third column, our method can also provide better detection results. For a more comprehensive analysis of our method, we present failure cases in the last column. For example, for the smaller aeroplane in Figure 7a, it is difficult for our method to learn its instance representation. Our method also fails to detect the highly overlapping sheep and distant little sheep in Figure 7b.

## 5. Conclusions

In this paper, we propose an instance-level contrastive learning (ICL) framework to guide the weakly supervised detector to learning entire instance representations by constructing instance correlations with other images. To store diverse object instance representations in a memory bank, we propose an instance-diverse memory updating (IMU) algorithm. With the help of memory, we further propose a memory-aware instance mining (MIM) algorithm to effectively mine object instances. To alleviate the imbalance of positive and negative proposals, we propose a memory-aware proposal sampling (MPS) algorithm. We conduct extensive experiments on PASCAL VOC2007 and VOC2012 datasets to verify the effectiveness of our method.

Our proposed method mines object instance representations from other images and stores them in a memory bank to guide instance learning on the current image. If the memory contains noisy representations, it will make the learned object instances inaccurate. The performance of weakly supervised detectors is also limited by the quality of the stored representations. In order to mine more reliable instance representations, our future studies will explore contextual information of region proposals or segmentation information of images to perceive object boundaries and locate object instances accurately. 

## Figures and Tables

**Table 1:** Comparison with other methods on Pascal VOC2007 *test* set. ${}^{★}$ means our baseline.

Method	aer	bik	bir	boa	bot	bus	car	cat	cha	cow	tab	dog	hor	mot	per	pla	she	sof	tra	tv	mAP
WSDDN [20]	39.4	50.1	31.5	16.3	12.6	64.5	42.8	42.6	10.1	35.7	24.9	38.2	34.4	55.6	9.4	14.7	30.2	40.7	54.7	46.9	34.8
Kantorov et al. [14]	57.1	52.0	31.5	7.6	11.5	55.0	53.1	34.1	1.7	33.1	49.2	42.0	47.3	56.6	15.3	12.8	24.8	48.9	44.4	47.8	36.3
OICR [15] ${}^{★}$	58.0	62.4	31.1	19.4	13.0	65.1	62.2	28.4	24.8	44.7	30.6	25.3	37.8	65.5	15.7	24.1	41.7	46.9	64.3	62.6	41.2
PCL [26]	54.4	69.0	39.3	19.2	15.7	62.9	64.4	30.0	25.1	52.5	44.4	19.6	39.3	67.7	17.8	22.9	46.6	57.5	58.6	63.0	43.5
C-MIL [21]	62.5	58.4	49.5	32.1	19.8	70.5	66.1	63.4	20.0	60.5	52.9	53.5	57.4	68.9	8.4	24.6	51.8	58.7	66.7	63.5	50.5
Lin et al. [22]	55.6	67.0	45.8	27.9	21.1	69.0	68.3	70.5	21.3	60.2	40.3	54.5	56.5	70.1	12.5	25.0	52.9	55.2	65.0	63.7	50.1
TS${}^{2}$C [18]	59.3	57.5	43.7	27.3	13.5	63.9	61.7	59.9	24.1	46.9	36.7	45.6	39.9	62.6	10.3	23.6	41.7	52.4	58.7	56.6	44.3
WS-JDS [16]	52.0	64.5	45.5	26.7	27.9	60.5	47.8	59.7	13.0	50.4	46.4	56.3	49.6	60.7	25.4	28.2	50.0	51.4	66.5	29.7	45.6
SDCN [19]	59.8	67.1	32.0	34.7	22.8	67.1	63.8	67.9	22.5	48.9	47.8	60.5	51.7	65.2	11.8	20.6	42.1	54.7	60.8	64.3	48.3
Yang et al. [17]	-	-	-	-	-	-	-	-	-	-	-	-	-	-	-	-	-	-	-	-	50.6
SLV [27]	65.6	71.4	49.0	37.1	24.6	69.6	70.3	70.6	30.8	63.1	36.0	61.4	65.3	68.4	12.4	29.9	52.4	60.0	67.6	64.5	53.5
D-MIL [25]	60.4	71.3	51.1	25.4	23.8	70.4	70.3	71.9	25.2	63.4	42.6	67.1	57.7	70.1	15.5	26.6	58.7	63.3	66.9	67.6	53.5
ICL	61.9	73.0	44.0	33.3	32.9	75.3	74.7	73.8	2.6	70.6	62.0	60.8	72.2	71.3	26.0	25.4	57.3	57.7	72.7	60.9	**55.4**

**Table 2:** Comparison with other methods on Pascal VOC2007 *trainval* set. ${}^{★}$ means our baseline.

Method	aer	bik	bir	boa	bot	bus	car	cat	cha	cow	tab	dog	hor	mot	per	pla	she	sof	tra	tv	mean
WSDDN [20]	65.1	58.8	58.5	33.1	39.8	68.3	60.2	59.6	34.8	64.5	30.5	43.0	56.8	82.4	25.5	41.6	61.5	55.9	65.9	63.7	53.5
Kantorov et al. [14]	83.3	68.6	54.7	23.4	18.3	73.6	74.1	54.1	8.6	65.1	47.1	59.5	67.0	83.5	35.3	39.9	67.0	49.7	63.5	65.2	55.1
OICR [15] ${}^{★}$	81.7	80.4	48.7	49.5	32.8	81.7	85.4	40.1	40.6	79.5	35.7	33.7	60.5	88.8	21.8	57.9	76.3	59.9	75.3	81.4	60.6
PCL [26]	79.6	85.5	62.2	47.9	37.0	83.8	83.4	43.0	38.3	80.1	50.6	30.9	57.8	90.8	27.0	58.2	75.3	68.5	75.7	78.9	62.7
C-MIL [21]	-	-	-	-	-	-	-	-	-	-	-	-	-	-	-	-	-	-	-	-	65.0
Lin et al. [22]	-	-	-	-	-	-	-	-	-	-	-	-	-	-	-	-	-	-	-	-	67.2
TS${}^{2}$C [18]	84.2	74.1	61.3	52.1	32.1	76.7	82.9	66.6	42.3	70.6	39.5	57.0	61.2	88.4	9.3	54.6	72.2	60.0	65.0	70.3	61.0
WS-JDS [16]	82.9	74.0	73.4	47.1	60.9	80.4	77.5	78.8	18.6	70.0	56.7	67.0	64.5	84.0	47.0	50.1	71.9	57.6	83.3	43.5	64.5
SDCN [19]	85.8	83.1	56.2	58.5	44.7	80.2	85.0	77.9	29.6	78.8	53.6	74.2	73.1	88.4	18.2	57.5	74.2	60.8	76.1	79.2	66.8
SLV [27]	84.6	84.3	73.3	58.5	49.2	80.2	87.0	79.4	46.8	83.6	41.8	79.3	88.8	90.4	19.5	59.7	79.4	67.7	82.9	83.2	71.0
D-MIL [25]	81.3	82.0	72.7	48.9	42.0	80.2	86.1	78.5	43.9	80.2	42.2	76.5	68.7	91.2	32.7	56.0	81.4	69.6	78.7	79.9	68.7
ICL	85.3	88.9	65.5	57.5	57.4	86.0	90.7	85.8	15.1	88.7	78.0	74.4	89.2	93.5	39.2	57.6	88.5	71.6	86.2	80.1	**74.0**

**Table 3:** Comparison with other methods on Pascal VOC 2012 dataset. ${}^{★}$ means our baseline.

Method	mAP	CorLoc
Kantorov et al. [14]	35.3	54.8
OICR [15] ${}^{★}$	37.9	62.1
PCL [26]	40.6	63.2
C-MIL [21]	46.7	67.4
Lin et al. [22]	45.3	67.1
TS${}^{2}$C [18]	40.0	64.4
WS-JDS [16]	39.1	63.5
SDCN [19]	43.5	67.9
SLV [27]	49.2	69.2
D-MIL [25]	49.6	70.1
ICL	**50.1**	**70.4**

**Table 4:** The contribution of each component of our method. Both PASCAL metrics and COCO metrics are applied.

Method	PASCAL Metrics		COCO Metrics					
	mAP	CorLoc	AP	AP${}_{50}$	AP${}_{75}$	AP${}_{S}$	AP${}_{M}$	AP${}_{L}$
Baseline	41.2	60.6	14.9	37.0	10.7	1.8	8.6	19.4
ICL	**55.4**	**74.0**	**20.8**	**48.8**	**14.4**	2.3	10.3	**27.1**
ICL w/o IMU	53.6	72.9	19.8	46.8	14.2	2.8	10.1	25.9
ICL w/o MIM	49.7	69.6	18.0	43.1	12.5	2.1	8.9	23.8
ICL w/o MPS	52.9	71.4	19.5	46.7	13.5	**3.3**	**10.5**	25.0

**Table 5:** The effect of the removal coefficient 1/λ.

1/λ	mAP	CorLoc
1/10	52.7	70.8
1/8	52.0	70.1
1/6	53.6	72.6
1/4	**55.4**	**74.0**
1/2	52.4	70.8

## Data Availability

The PASCAL VOC2007 and VOC2012 datasets can be available in the following link: http://host.robots.ox.ac.uk/pascal/VOC/.
